# Cryptosporidium Infection and Associated Risk Factors among Cattle in the Central Region of Ghana

**DOI:** 10.1155/2021/6625117

**Published:** 2021-04-04

**Authors:** Kwabena Dankwa, Patrick K. Feglo, Samuel V. Nuvor, Michael Aggrey-Korsah, Mohamed Mutocheluh

**Affiliations:** ^1^Department of Clinical Microbiology, Kwame Nkrumah University of Science and Technology, Kumasi, Ghana; ^2^Department of Microbiology and Immunology, University of Cape Coast, Ghana; ^3^Veterinary Service Directorate, Cape Coast, Ghana

## Abstract

*Cryptosporidium* species infects a wide number of animals including livestock all over the world. The current study was done to determine the prevalence and risk factors of *Cryptosporidium* infection among cattle in the Central Region of Ghana. Two hundred and eighty-seven (287) faecal samples were randomly collected from animals on eight cattle farms in four districts across two agroecological zones. A commercial enzyme-linked immunosorbent assay kit (CoproELISA, Savyon® Diagnostics Ltd., Israel) for *Cryptosporidium* was used in the detection of *Cryptosporidium* antigens in faecal samples. Characteristics of the animals such as age, sex, and location, as well as consistency of faecal samples, were collected. Pearson's chi-square or Fisher's exact test was used to determine the association between explanatory variables and *Cryptosporidium* infection while a logistic regression model was also used to determine the risk of infection. The overall prevalence of *Cryptosporidium* infection was 23.7% (95% CI, 18.7-28.6). Prevalence was significantly higher (*p* = 0.049) among cattle aged 12-month old and above compared to those under 12 months of age. Among the four districts in the study area, Cape Coast metropolis recorded a significantly higher prevalence (60.5%; CI, 49.3-71.8), (*p* < 0.001) compared to the other three. Furthermore, a significant association was observed between the consistency of faecal samples and *Cryptosporidium* infection (*p* = 0.042). The prevalence of *Cryptosporidium* infection was also significantly higher among cattle from the coastal savanna zone (26.9%; 95% CI, 21.0-32.8) compared to those from the semideciduous forest area (*p* = 0.017). Cattle in the forest zone had a lower risk of being infected with the parasite compared to those from the coastal savanna zone (OR 0.408; 95% CI, 0.182-0.915). In conclusion, *Cryptosporidium* was prevalent among cattle in the Central Region of Ghana. A higher prevalence of *Cryptosporidium* infection occurred in older animals and among animals in the coastal agroecological zone. The area of location and age of animals were identified as risk factors for *Cryptosporidium* infection in the Central Region of Ghana.

## 1. Introduction

*Cryptosporidium* species are common enteroparasites known to infect a number of vertebrates including fishes, birds, reptiles, mammals, and humans [1–3]. This coccidian parasite produces hardy oocysts which are ubiquitous in the environment [4–6]. The oocysts are ingested along with contaminated food or water and infect the gastrointestinal tract of the host [7, 8].

Currently, about 38 valid species and several genotypes have been identified, with cattle hosting 13 of them [9–11]. Among the livestock population, cattle are commonly infected by four main species: *Cryptosporidium parvum*, *C. bovis*, *C. andersoni*, and *C. ryanae* [12–14]. Infection in cattle may be inapparent or may present with severe morbidity. Cryptosporidiosis is common in neonatal calves and generally is characterized by diarrhoea, abdominal pain, anorexia, and loss of weight and retarded growth [12, 15].

The main diagnostic approach for many studies on *Cryptosporidium* in Ghana is by microscopy [16, 17]. Microscopic detection of *Cryptosporidium* oocysts involves the concentration of stool samples and modified Ziehl-Neelsen staining. This method is however laborious, time-consuming, of low sensitivity and requires the expertise of experienced microscopists. Only a handful of studies have utilized enzyme-linked immunosorbent assay (ELISA) for the detection of *Cryptosporidium* antigens in stool samples in Ghana [18]. The ELISA method, although expensive, is known to have better sensitivity compared to microscopy and relatively faster as many samples can be processed within a shorter time frame [19–21].

There are many reports of *Cryptosporidium* infections in livestock such as cattle worldwide [22–25]. In neighbouring Nigeria in the West African region, several studies of *Cryptosporidium* infections in cattle have been conducted [26–28]. Studies undertaken in Ghana regarding *Cryptosporidium* among livestock in general and cattle in particular are few although the prevalence of between 19.7% and 29% has been reported [16, 29, 30]. The only study of *Cryptosporidium* infection of livestock in the Central Region which included cattle, sheep, and goats as part of a larger study in Ghana reported a prevalence of 14.1% and 18.9% in the Komenda Edina Eguafo Abirem and Awutu Senya districts [30].

Cattle rearing in Ghana is done mainly by extensive and semi-intensive management systems which allow the animals to graze on natural pastures bringing them into close contact with farmland and water bodies [16]. Infective oocysts passed in the faeces of these animals contribute significantly to the contamination of the environment with potential for further transmission to other animals and humans [22, 31]. Despite the immense public health and veterinary importance of this parasite, there is limited data on the prevalence and risk factors for its infection across the Central Region of Ghana. The present study is aimed at determining the occurrence of *Cryptosporidium* infection and associated risk factors among cattle in the Central Region of Ghana.

## 2. Materials and Methods

### 2.1. Study Design/Area

This was a field-based, cross-sectional study carried out between November 2018 and June of 2019. This study was conducted in four districts which cut across the two agroecological zones of the Central Region of Ghana. The Central Region occupies a total area of 9,826 square kilometres of the country and is in the southern part. The two main agroecological zones in the region are the coastal savanna and the semideciduous forest. Climatically, the rainfall pattern in the region is bimodal with the major rainy season from April to July and minor from September to November, generally referred to as the wet season. The forest areas receive higher amounts of precipitation. The months of December to March form the dry season characterized by dusty dry harmattan winds.

**Figure 1:**
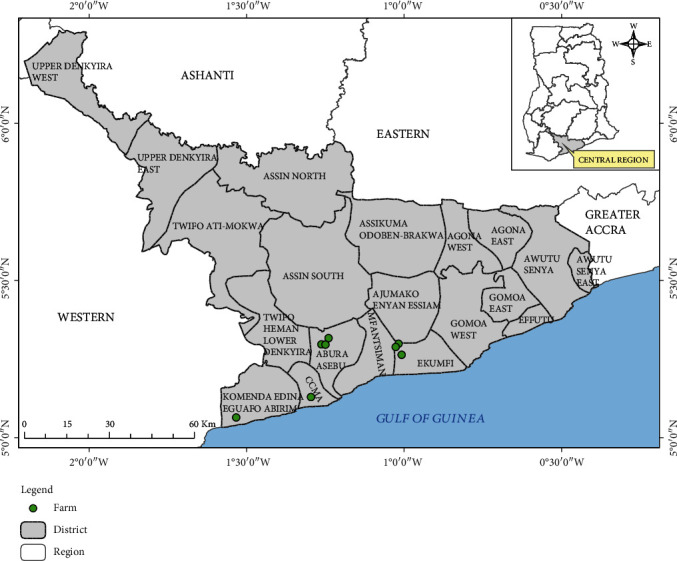
Map of the Central Region showing farm locations and districts where study samples were collected. Credit: Mr. Richard Adade, GIS and Remote Sensing Unit, Centre for Coastal Management, University of Cape Coast, Ghana.

Cattle rearing in the study area is done largely in the coastal ecological zone mainly by the semi-intensive system of management where the animals are led out by herders for grazing and watering, eventually returning to the kraals before the evening. There are some cattle farms in the forest ecological zones, although the herd sizes are small. The cattle breeds sampled for the current study included Zebu, Ndama, West African shorthorn, Sokoto Gudali, white Fulani, and crossbreeds. Cattle selected for the study were from 8 farms in the 4 districts, namely, Cape Coast (CCMA), Komenda Edina Eguafo Abirem, Abura Asebu Kwamankese, and Mfantseman as shown in Figure 1. The four districts were purposely chosen because they are noted for cattle production in the region. However, the selection of cattle farms for the study was done randomly from a list of farms in the districts provided by the regional veterinary office. Three farms were in the forest agroecological area of Abura Asebu Kwamankese, while the rest were in the coastal savanna zone of the other three districts. Cattle were selected randomly using systematic sampling as they were led out of their crash pens for grazing in the morning.

### 2.2. Study Population and Sample Size

Cattle of different ages (<12 months, 12-24 months, and >24 months) and sexes were selected randomly from eight (8) cattle farms in the study area. Using the average prevalence from previous studies, 19.7% and 27% in the southern part of Ghana [29, 30], *Cryptosporidium* prevalence of 24% was used for the sample estimation, done at 95% confidence interval and 5% margin of error using Cochran's formula [32] giving a minimum of 280 samples. However, a total number of 287 animals were involved in the study.

### 2.3. Sample Collection and Storage

A single fresh faecal sample was obtained from each animal. The samples were collected directly from the rectum of the animals with sterile disposable latex gloves. In a few cases, the animals were observed to defecate after which the faecal sample was collected immediately from the ground without contamination. Approximately 20-30 grams of fresh faecal material was collected and labelled with a unique identification number.

For each sample collected, information on farm location, sex, and age of the animal were also recorded. The samples were then placed in an icebox and transported to the laboratory for analysis. In the laboratory, the faecal samples were examined macroscopically for their consistency and then aliquoted into 2 ml tubes containing 10% formalin.

### 2.4. Laboratory Analysis

#### 2.4.1. ELISA

The presence or absence of *Cryptosporidium* antigens in the faecal samples was detected using a commercial enzyme-linked immunosorbent assay kit, “CoproELISA” *Cryptosporidium* by Savyon Diagnostics, Israel, according to the manufacturer's instructions. Briefly, about 0.15 g or 150 *μ*l of 10% formalin preserved faecal samples were homogenized in 400 *μ*l stool diluent in 2.0 ml Eppendorf tubes and vortexed. One hundred microlitres of the positive control was added to a well of the microtiter plate coated with anti-cryptosporidium antibodies, while two wells of the negative control diluent each received 100 *μ*l. One hundred microlitres of faecal samples already homogenized in the diluent was added to the wells and incubated at 37°C for 1 hour. The plate was washed five times with 300 *μ*l of washing buffer followed by the addition of 100 *μ*l of horseradish peroxidase (HRP) conjugate and then incubated at 37°C for another 1 hour.

After washing with buffer for 5 times, 100 *μ*l of tetramethylbenzidine (TMB) substrate was added and incubated for 15 minutes, followed by the addition of 100 *μ*l of stop solution to the wells. The plate was then read at 450 nm using the ELISA reader (Thermo Scientific Multiskan EX). Sample absorbance was compared to a cut-off value obtained from the average absorbance in the negative control wells to determine the presence or absence of cryptosporidial antigens. Cryptosporidial antigens were detected (positive) if the sample absorbance was equal or higher than the cut-off value and nondetectable (negative) when sample absorbance was lower than the cut-off value.

### 2.5. Statistical Analysis

Data was entered into IBM SPSS Statistics for Windows, version 25.0 (Armonk, NY: IBM Corp.) for statistical analysis. Summary of the data was obtained using descriptive statistics and presented frequencies and proportions with their confidence intervals. The associations between *Cryptosporidium* infection and other explanatory variables such as potential risk factors were assessed using Pearson's chi-square or Fisher's exact test where appropriate. We also performed logistic regression to determine the risk of *Cryptosporidium* infection in cattle. The model consisted of three independent variables (agroecological location, age category, and faecal consistency). Odds ratios at 95% confidence intervals were calculated to determine the strength of the association between the explanatory variables and *Cryptosporidium* infection. All through the analysis, *p* < 0.05 was considered statistically significant.

## 3. Results

The overall prevalence of *Cryptosporidium* infection among the cattle population in this study by antigen detecting ELISA was 68/287, representing 23.7% (95% CI, 18.7-28.6).

Female animals formed 80.8% of the sampled population. Majority of faecal samples collected in the study were nondiarrhoeic (82.9%) while diarrhoeic samples constituted 17.1%. In addition, the majority of animals in this study (62.0%) were more than 24 months old (Table 1).

The prevalence of *Cryptosporidium* antigens in faecal samples was higher among female cattle, 24.1% (95% CI, 18.6-29.7) compared to that of males, 21.8% (95% CI, 10.6-33.1) even though the difference was not significant (*p* = 0.716). Among the three age categories of cattle involved in the study, the prevalence of *Cryptosporidium* was 27.3% (95% CI, 13.6-41.0) and 27.0% (95% CI, 20.4-33.5) among animals in the 12-24-month and over 24-month groups, respectively, compared to the 12.3% (95% CI, 4.1-20.5) found in animals under 12 months of age. The difference in this group was statistically significant (*p* = 0.049). Also, while a higher number, 26.1% (95% CI, 20.4-31.7) of *Cryptosporidium* infection was recorded among cattle with nondiarrhoeic samples, and only 12.2% (95% CI 2.7-21.8) of diarrhoeic samples tested positive for *Cryptosporidium* antigens; the difference was statistically significant (*p* = 0.042), as shown in Table 1.

*Cryptosporidium* was detected among cattle in all four districts and the two agroecological zones. The majority (77.7%) of the cattle were from the coastal savanna agroecological zone, while the Komenda Edina Eguafo Abirem municipality had the highest number of cattle (28.9%) participating in the study compared to the other districts (Table 2).

Generally, there was an association between the location of cattle and *Cryptosporidium* infection. The prevalence of the parasite was significantly higher among cattle from the coastal agroecological zone, 26.9% (95% CI, 21.0-32.8) compared to those from the semideciduous forest zone, 12.5% (95% CI, 4.2-20.8) (*p* = 0.017). Similarly, cattle in the Cape Coast metropolis recorded the highest prevalence of *Cryptosporidium* infection, 60.5% (95% CI, 49.3-71.8), while Komenda Edina Eguafo Abirem recorded the lowest level of infection, 6.0% (95% CI, 0.8-11.3). There was a significant difference in the prevalence of the parasite among the district/municipalities/in the study area (*p* < 0.001) as shown in Table 2.

The location of cattle and age emerged as the strongest predictors of *Cryptosporidium* infection while faecal consistency did not. The results from the multivariate analysis showed that animals aged 24 months old and above presented a higher risk of infection by *Cryptosporidium* species (OR = 2.64; 95% CI, 1.163-6.000; *p* = 0.20). Similarly, an odds ratio of 0.408 (95% CI, 0.182-0.915) was obtained for cattle from the semideciduous forest agroecological zone compared to those from the coastal savanna zone as presented in Table 3.

## 4. Discussion

*Cryptosporidium* is a parasite known to infect the gastrointestinal tracts of both humans and animals including cattle. Although zoonotic species of the parasite can be found in cattle with possibility for transmission to humans, most studies in Ghana on the parasite have focused on the human population. The current study is one of the few to address the occurrence of *Cryptosporidium* species among cattle in the southern part of Ghana [16, 29, 30]. It involved the use of enzyme-linked immunosorbent assay (ELISA) for the detection of *Cryptosporidium* antigens in faecal samples as well as the determination of some risk factors for infection of cattle with the parasite.

The overall prevalence of *Cryptosporidium* in the current study was 23.7% and which is within the range of 1%-86% reported in some countries in Africa among cattle [33, 34]. A similar study in the coastal and forest transitional zones of the Greater Accra region and another one in catchment area of the Kpong and Weija lakes in the Eastern and Greater Accra regions using the Ziehl-Neelsen modified acid-fast staining technique and microscopy reported prevalence rates of 29% and 19.7%, respectively [16, 29]. In addition, a recent study in the coastal savanna zones of the Central, Greater Accra, and Volta regions which used molecular methods to characterize *Cryptosporidium* and *Giardia* among farmers and their livestock recorded a *Cryptosporidium* prevalence rate 26.5% among cattle [30].

The prevalence obtained in this study is comparable to that of a study done in Nigeria using the Kinyoun acid-fast staining method in which among the 406 cattle sampled, a prevalence of 23.4% was reported [26]. In the Ogun state of Nigeria, one study involving the detection of *Cryptosporidium* coproantigens recorded a higher prevalence of 37.5% [27] than the present study. The prevalence rate for the current study was however higher than a similar study in Addis Ababa, Ethiopia, which reported a rate of 18.6% using both modified Ziehl-Neelsen staining and molecular techniques [22]. A study in Aizawl district in India using both modified Ziehl-Neelsen and molecular detection [35] and one other in Malaysia peninsular using only molecular detection method for Cryptosporidium [15] also recorded rates of 13% and 12.5%, respectively, which were lower compared to the present study. The varying prevalence of *Cryptosporidium* infection observed among cattle in different studies may be attributed to several reasons including season of sampling, the geographic and ecological location of the study areas, laboratory techniques employed in the detection of the parasite, type of animal management system practiced by the farmers, the breed of the animals, and hygiene status of the farms or kraals as well as the climate of the area under study [15, 16, 29, 36].

Our data showed that *Cryptosporidium* infection was significantly higher among cattle aged 12 months and above compared to animals that were 12 months old and younger. This report is contrary to many other reports which suggested that *Cryptosporidium* infections were higher in younger animals than adults [35, 37, 38]. This is quite unexpected since younger animals, especially calves, are known to have reduced immunity and are therefore susceptibly prone to infection which normally leads to diarrhoea and other manifestations. It may also be because the calves were less exposed to the sources of contamination. It is common knowledge that there is an age-related association regarding the four main species of the parasite in cattle. *Cryptosporidium andersoni* was reported to prefer older animals, while *Cryptosporidium parvum*, *Cryptosporidium ryanae*, *and Cryptosporidium bovis* were predominant in younger calves [12, 39]. However, the method employed in this study (antigen detection by ELISA) did not allow us to determine the species of the parasite responsible for the high prevalence in adult cattle. The high prevalence of the parasite in older animals is quite worrying since the cattle in the study area are managed on a semi-intensive basis which allows them to graze on the open fields outside their kraals with the possibility of contaminating the environment and water bodies in the area. Adult cattle could also serve as sources of infection for preweaned and weaned calves and other susceptible livestock and animals in grazing areas, increasing the potential for transmission of the parasite.

Prevalence of *Cryptosporidium* species was also higher in female cattle than in their male counterparts, although the difference was not statistically significant (*p* = 0.716). This report is comparable to a study done in the coastal and forest savannah transition zone of the Greater Accra region of Ghana [36] and in the Aizawl district in India [35] and another one among native cattle herds in Nigeria [37]. It is however contrary to a study in Nigeria which recorded a significantly higher prevalence of *Cryptosporidium* among male animals [37, 40].

Regarding the consistency of faecal samples, *Cryptosporidium* prevalence was significantly higher among cattle with nondiarrhoeic samples compared to those with diarrhoea. This is an interesting observation as *Cryptosporidium* infection is normally prevalent among young animals and is also associated with loose or diarrhoeic stools [29]. A report from the Oyo state in Nigeria using ELISA and molecular methods found no significant difference in the prevalence of the parasite between nondiarrhoeic and diarrhoeic stool samples of cattle [37]. Also, the current finding is contrary to other studies in India and Ethiopia which reported a significantly higher prevalence among animals with diarrhoeic samples compared to those with nondiarrhoeic samples [35, 36]. The observation of a high number of diarrhoeic samples from adult animals compared to young ones may be because young animals formed a smaller proportion of sample in the study. In addition, the aetiology of diarrhoea among the animals could be from other causes than *Cryptosporidium*, which was not investigated in this study.

The prevalence of *Cryptosporidium* in cattle was significantly higher among cattle in the coastal agroecological zone than the semideciduous forest zone and also compared well to studies done in other parts of Southern Ghana [29, 30]. This finding, however, contrasts with the previous study in which the drier coastal savanna areas recorded a lower prevalence compared to the more vegetated coastal savanna-forest transitional zone [16]. In Ghana, the coastal agroecological zone has suitable vegetation such as grassland for cattle rearing and so has a higher population of cattle compared to the forest zone. It may also be speculated that the low prevalence of the parasite in the forest agroecological zone may be due to the resistance offered by the breeds of cattle in that zone to *Cryptosporidium* infection compared to cattle in the coastal areas as well as the different grazing conditions.

*Cryptosporidium* was recorded in all four districts in this study, indicating the widespread occurrence of the parasite. *Cryptosporidium* prevalence was higher in districts or municipalities where cattle farms were situated in or near the cities/towns. It was observed in the current study that cattle from the Cape Coast metropolis recorded the highest prevalence of the parasite. It must be noted that the study sites in Cape Coast and Mfantseman districts were urban and periurban localities, respectively, which have their accompanying challenges of sanitation and waste disposal. We also speculate here that it could also be as a result of the increased interactions between the animals and human activities in these two areas which are urban and periurban in nature compared to the more rural setting of the cattle in the forest zone in this study. *Cryptosporidium* oocysts have the capacity to survive in the environment for long periods, and the contamination of pasture lands, grazing areas, and water bodies in these areas coupled with the semiextensive system of cattle management is likely to facilitate and promote the transmission of the parasite.

ELISA was the only diagnostic technique used to detect *Cryptosporidium* in the current study and was unable to distinguish between species of the parasite. The inability of the test to detect species of the parasite in this study did not allow us to understand the transmission routes and possible zoonotic potential. In future studies, other tests such as the polymerase chain reaction and restriction fragment length polymorphism or sequencing would be required for the identification of the parasite species and genotypes and in other parts of Southern Ghana to enable a broader understanding of the occurrence of the parasite.

While ELISA has high sensitivity in the detection of *Cryptosporidium* infection, low levels of antigens in samples may go undetected and will be reported as negative. This together with the intermittent shedding of the parasite in the host may lead to an underestimation of the prevalence of the parasite. In addressing this shortcoming, obtaining repeated samples from participants would have been the best option, but this was not done in the current study. Besides, the current study also failed to capture the seasonal variation in the prevalence of the parasite as sampling was for only part of the year.

## 5. Conclusions

*Cryptosporidium* infection is common among cattle across the two agroecological zones of the Central Region of Ghana with an overall prevalence of 23.7%. Higher prevalence of the parasite occurs in the coastal savanna ecological zone than the semideciduous forest zone. The parasite is also present among cattle in all four districts involved in the study with varying levels of prevalence. The agroecological area of location and age of cattle are risk factors for infection of cattle with the parasite. There is potential for the spread and transmission of the parasite in the region among cattle, and so, further evaluation is required to determine the prevailing species, genotypes, and subtypes of *Cryptosporidium* to give a better understanding of the transmission dynamics and the zoonotic potential of the parasite.

## Figures and Tables

**Table 1:** Occurrence of *Cryptosporidium* infection in cattle by sex, age, and faecal consistency.

Variable	Number of samples (%)	Number of positives (%)	95% CI	*p* value
Sex				
Male	55 (19.2)	12 (21.8)	10.6-33.1	0.716
Female	232 (80.8)	56 (24.1)	18.6-29.7	
Age category				
<12 months	65 (22.7)	8 (12.3)	4.1-20.5	0.049^∗^
12-24 months	44 (15.3)	12 (27.3)	13.6-41.0	
>24 months	178 (62.0)	48 (27.0)	20.4-33.5	
Faecal consistency				
Nondiarrhoeic	238 (82.9)	62 (26.1)	20.4-31.7	0.042^∗^
Diarrhoeic	49 (17.1)	6 (12.2)	2.7-21.8	

^∗^Statistically significant at *p* < 0.05. CI: confidence interval.

**Table 2:** Occurrence of *Cryptosporidium* infection in cattle by location of farm.

Farm location	Number of samples (%)	Number of positives (%)	95% CI	*p* value
Agroecological zone				
Coastal savanna	223 (77.7)	60 (26.9)	21.0-32.8	0.017^∗^
Semideciduous forest	64 (22.3)	8 (12.5)	4.2-20.8	
District/municipality/metropolis				
Mfantseman	64 (22.3)	9 (14.1)	5.3-22.8	
Cape Coast	76 (26.5)	46 (60.5)	49.3-71.8	<0.001^∗^
Komenda Edina Eguafo Abirem	83 (28.9)	5 (6.0)	0.8-11.3	
Abura Asebu Kwamankese	64 (22.3)	8 (12.5)	4.2-20.8	

^∗^Statistically significant at *p* < 0.05. CI: confidence interval.

**Table 3:** Risk factors for *Cryptosporidium* infection among cattle in the Central Region.

Variable	Prevalence (%)	Odds ratio (95% CI)	*p* value
Age category			
<12 months	12.3	1	
12-24 months	27.3	2.45 (0.895-6.708)	0.81
>24 months	27.0	2.64 (1.163-6.000)	0.20^∗^
Faecal consistency			
Nondiarrhoeic	26.1	1	
Diarrhoeic	12.2	0.413 (0.165-1.037)	0.60
Agroecological zone			
Coastal savanna	26.9	1	
Semideciduous forest	12.5	0.408 (0.182-0.915)	0.030^∗^

^∗^Statistically significant at *p* < 0.05. CI: confidence interval.

## Data Availability

The data for this work is available upon request from the corresponding author.
